# Validity of the motivated strategies for learning questionnaire in Saudi Arabia

**DOI:** 10.5116/ijme.5bec.81cf

**Published:** 2018-11-30

**Authors:** Salman Bin Dayel, Abdurrahman Al Diab, Adel Abdelaziz, Amira Farghaly, Ahmed Al Ansari

**Affiliations:** 1 Department of Dermatology, College of Medicine, Prince Sattam Bin Abdulaziz University, Saudi Arabia; 2 Department of Internal Medicine, College of Medicine, King Saud University, Saudi Arabia; 3 Department of Medical Education, College of Medicine, Prince Sattam Bin Abdulaziz University, Saudi Arabia; 4 Training and Education Department, Bahrain Defense Force, Bahrain

**Keywords:** Motivation, CFA, scale development, Saudi Arabia

## Abstract

**Objectives:**

To assess the construct validity and reliability of the motivation section of the Motivated Strategies for Learning Questionnaire in Saudi Arabia.

**Methods:**

A cross-sectional study using the Motivated Strategies for Learning Questionnaire (MSLQ) was conducted. The MSQL has essentially two sections: a motivation section and a learning strategies section.  The motivation section, which consists of 31 items, was used.  A total of 146 medical students who were all male completed the questionnaire. Confirmatory factor analysis was used to test the hypothesised factor structure, and to identify the validity and reliability of the motivation section of the MSQL.

**Results:**

A selected group of fit statistics showed that the hypothesised model did not fit the sample data fairly well.  The Intrinsic Goal Orientation subscale consisted of 4 items (α = 0.75), the Extrinsic Goal Orientation subscale consisted of 4 items (α = 0.78), the Task Value subscale consisted of 6 items (α =0.86), the Control of Learning Beliefs consisted of 4 items (α =0.78), the Self-Efficacy for Learning and Performance consisted of 8 items (α =0.89), and the Test Anxiety consisted of 5 items (α =0.77).

**Conclusions:**

It is concluded that the hypothesised model did not fit the data well. This may suggest that the motivation section of the MSQL may not work for Saudi Arabian students. However, this could be due to the fact that the sample data available on this study did not represent female students. Further work is required to establish this. Limitations of the study are discussed.

## Introduction

Pintrich and colleagues^1^ highly emphasised the relationship between motivation and cognition in student performance and learning. It is well documented that there is a relationship between motivation and student performance.^2^^-^^4^ This suggests that if we measure the academic motivation of students using a specific tool, we will able to improve the learning strategies of students.^5^ Pintrich and colleagues have developed the Motivated Strategies for Learning Questionnaire (MSLQ) in order to measures “students’ motivational orientations and their use of different learning strategies for a specific college course.^1^

The MSLQ is based on a general cognitive view of motivation and learning strategies in which the student is an active information processor, and beliefs and cognitions mediate instructional input.^1^ The questionnaire consisted of two sections. The first section was designed to measure the academic motivation used by students. The second section was intended to measure the types of learning strategies. Since these sections measure two different constructs, therefore each section can be used separately or together. Therefore, based on the purpose of this study, researchers can use one of these sections or use both sections together.^1^^,^^5^   As the purpose of this study is to fit a model for the academic motivation of medical students, the section of motivation is used.

The academic motivation section of the MSLQ is based on the social-cognitive model of motivation, which consisted of three general motivational constructs: expectancy, value and affect.^6^^,^^7^ The expectancy construct focuses on students’ beliefs that they can accomplish a task, and it consists of two subscales. The first subscale is the control of learning beliefs (the belief that outcomes depend on effort and ability rather than on external factors), and the second is self-efficacy (the confidence that one’s abilities are sufficient to succeed). The construct of value focuses on the student’s reasons for engaging in an academic task and consisted of three subscales. The first subscale is intrinsic goal orientation (a focus on learning and mastery), the second subscale is extrinsic goal orientation (a focus on grades and approval from others), and the third subscale is task value beliefs (a judgement of how interesting, useful and essential the student finds the course content).^8^ The last construct is the construct of affect, and it includes responses to a test anxiety scale, which addresses student worries.^8^

The MSLQ, either in its entirety or the form of selected subscales, has been used in many different ways to improve learning.  For instance,  it has been used to measure the nature of motivation across different course subjects,^9^^-^^13^ and different populations.^5^^, ^^14^ It has also been used to measure the motivational effects of various aspects of teaching strategies and course structures.^15^^-^^18^ The MSLQ is a reliable and valid questionnaire for measuring academic motivation in some different countries with different cultures, including Estonia, Iran, Turkey, Pakistan, Portugal, Spain, China and America.^5^^,^^19^^-^^23^ It has also been used to improve education for undergraduate medical students and other healthcare students.^2^^-^^5^  Therefore, the questionnaire can be used  for different cultures to monitor and enhance the quality of educational strategies.

However, no previous studies have investigated the hypothesised model of the academic motivation section among Saudi Arabian medical students. Since social and cultural issues can be threats to the validity of test scores,^24^ one may argue that the MSLQ might perform differently among Saudi Arabian medical students who their culture is different with Western culture.  If this is the case, the MSLQ is required to be revised.^25^ Therefore, the aim of this study is to shine new light on these debates through an investigation of the hypothesised structure of the academic motivation section of the MSLQ^1^ among Saudi Arabian medical students.  A further aim is to provide reliability evidence for the subscales of the academic motivation of the MSLQ.

### Methods

### Study design and participants

A cross-sectional study was used to investigate the hypothesised model of the motivation section of the MSLQ. To achieve this, a total of 146 medical students in the University of Prince Sattam Bin Abdulaziz was recruited using a convenience sampling strategy. The University has recruitment targets for male students. For that reason, all of the participants of the study were male. They were in the second to the sixth year of their studies.

### Instrument

The motivation section of the MSLQ was used in this study includes 31 items answered on a seven-point Likert–type scale (1= “not at all true for me”, 7= “very true for me”), see Appendix 1. A student’s score is based on each subscale by summing the number of items and taking the average.^1^ The motivation section has six subscales used to represent numerous aspects of the students’ goals, and values for a particular course (e.g., students’ beliefs about their success to succeed in a particular course) and is a well-respected questionaire with satisfactory psychometric properties.^1^^, ^^26^^-^^28^ 

### Procedure

Prior to undertaking the investigation, ethical clearance was obtained from the Research Ethical Committee in the University of Prince Sattam Bin Abdulaziz, College of Medicine, Saudi Arabia. In order to protect research participants, the confidentiality and anonymity of participants and their data were preserved.  To collect data in a professional manner, we sent the questionnaire to the department of education at the University in order to print and distribute our survey questionnaires to the students. To gather an adequate sample size, the data were collected in different places. For example, some students completed the questionnaires in class time, and other students completed the questionnaire in the department of education.

### Statistical analysis

Confirmatory Factor Analysis of the responses was performed using the statistical packages SPSS and AMOS (version 22). This procedure was used both to measure the construct validity of the academic motivation section of the MSLQ and to test the fit of the hypothesised model to the sample data. To evaluate the construct validity of the questionnaire and assess whether the data fit the empirical model, a CFA was performed.  The following fit statistics were used to identify the model fit: the chi-square statistic ( a p-value higher than  0.01 suggests that the fit of the data to the hypothesised model is entirely adequate ).^31^ The standardized root mean square residual (SRMR) ( values higher than 0.09 suggests the sample data fit the model).^32^ The Tucker-Lewise Index (TLI) ( a value  ≥ 0.95 suggests the data adequately fit the model).32 The root mean square error of approximation (RMSEA) ( values < 0.06  suggests the initially hypothesised model fits the data well).^32^ The comparative fit index (CFI) ( a value ≥ 0.90 suggests acceptable model fit).^33^  Besides, the internal consistency reliability of the scale scores was measured using Cronbach’s alpha. An alpha of 0.70 is acceptable score reliability.^34^ The multiple imputation techniques was used to replace missing values prior to analysis which is used for structural equation modelling.^29^^,^^30^ Finally, the path diagrams were drawn to display the hypothesised model of the academic motivation section of the MSLQ.

## Results

**Figure 1:**
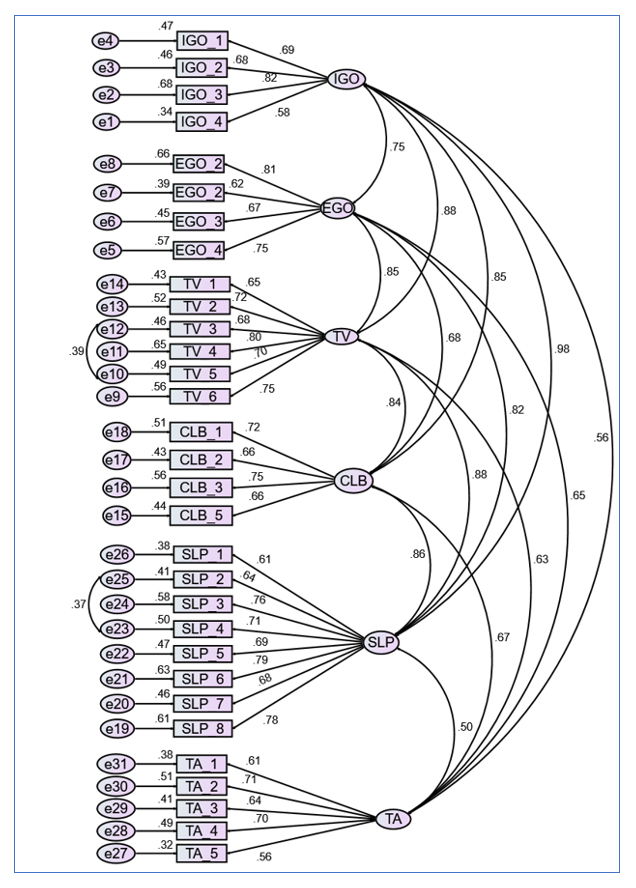
The six- factor model of the academic motivation section of the MSLQ - NOTE: IGO = Intrinsic Goal Orientation; EGO = Extrinsic Goal Orientation; TV= Task Value; CLB= Control of Learning Beliefs; SLP= Self-Efficacy for Learning and Performance; TA= Test Anxiety.

Table 1 shows the mean and standard deviation for each item within each subscale. As can be seen from the Table, Cronbach’s alphas for the subscales are satisfactory, a range from 0.75 to 0.89.  The hypothesised six-factor model of the academic motivation of the MSLQ is described graphically in Figure 1. Circles represent latent variables and rectangles represent measure variables.

**Table 1:** Means, standard deviations and reliability, (N=146)

Item	Mean	SD
Intrinsic Goal Orientation		
1. In a class like this, I prefer course material that really challenges me, so I can learn new things.	4.12	1.73
2. In a class like this, I prefer course material that arouses my curiosity, even if it is difficult to learn.	4.15	1.75
3. The most satisfying thing for me in this course is trying to understand the content as thoroughly as possible	4.40	1.71
4. When I have the opportunity in this class, I choose course assignments that I can learn from even if they don't guarantee a good grade	3.70	1.81
Reliability	0.75	
Extrinsic Goal Orientation		
1. Getting a good grade in this class is the most satisfying thing for me right now.	4.40	1.95
2. The most important thing for me right now is improving my overall grade point average, so my main concern in this class is getting a good grade	4.59	1.96
3. If I can, I want to get better grades in this class than most of the other students	4.72	2.02
4. I want to do well in this class because it is important to show my ability to my family, friends, employer, or others	4.08	1.96
Reliability	0.78	
Task Value		
1. I think I will be able to use what I learn in this course in other courses.	4.53	1.94
2. It is important for me to learn the course material in this class.	4.84	1.96
3. I am very interested in the content area of this course.	4.08	1.74
4. I think the course material in this class is useful for me to learn.	4.23	1.84
5. I like the subject matter of this course.	3.62	1.85
6. Understanding the subject matter of this course is very important to me	4.32	1.91
Reliability	0.867	
Control of Learning Beliefs		
1. If I study in appropriate ways, then I will be able to learn the material in this course.	4.64	1.98
2. It is my own fault if I don't learn the material in this course	3.95	1.87
3. If I try hard enough, then I will understand the course material	4.57	1.76
4. If I don't understand the course material, it is because I didn't try hard enough	3.79	1.89
Reliability	0.78	
Self-Efficacy for Learning and Performance		
1. I believe I will receive an excellent grade in this class.	3.81	1.76
2. I'm certain I can understand the most difficult material presented in the readings for this course	3.91	1.79
3. I'm confident I can understand the basic concepts taught in this course.	4.71	1.91
4. I'm confident I can understand the most complex material presented by the instructor in this course.	4.10	1.78
5. I'm confident I can do an excellent job on the assignments and tests in this course	4.34	1.67
6. I expect to do well in this class.	4.37	1.80
7. I'm certain I can master the skills being taught in this class.	4.19	1.77
8. Considering the difficulty of this course, the teacher, and my skills, I think I will do well in this class	4.30	1.84
Reliability	0.89	
Test Anxiety		
1. When I take a test, I think about how poorly I am doing compared with other students.	3.44	2.04
2. When I take a test I think about items on other parts of the test I can't answer	3.88	1.76
3. When I take tests I think of the consequences of failing.	3.47	2.08
4. I have an uneasy, upset feeling when I take an exam.	4.09	1.83
5. I feel my heart beating fast when I take an exam.	3.47	1.98
Reliability	0.77	

The single-headed arrowsrepresent the factor loadings (regression), and the bidirectional curved arrows represent factor correlations (factor covariance for unstandardized solutions). The path diagram also shows relationships among variables. The numbers “1” in the diagram reflect that the regression coefficient has been fixed to 1. As we hypothesised a six-factor model is confirmed in the academic motivation of the MSLQ, the model was tested using fit statistics.  The results of fit statistics for the one-factor model and the six- factor model are presented in Table 2. The values for the one-factor model indicate a poor fit between the model and the observed data. For the six-factor model, the only value that fits the model is SRMR which is less than 0.009. Taken together, these values suggest that both models do not fit our data set.

**Table 2:** Results of the confirmatory factor analysis (N= 146)

Model	Chi squared test	SRMR	RMSEA	CFI	TLI
One-factor model	*χ*^2^(434) = 1260.408, p<0.001	0.0831	0.115	0.699	0.678
Six –factor model	*χ*^2^(417) = 1013.35, p<0.001	0.07	0.100	0.779	0.758
Desired values	p>0.001	<0.09	<0.06	≥0.90	≥ 0.95

## Discussion

This study set out with the aim of assessing the academic motivation section of the MSLQ, using the CFA approach, in order to monitor and enhance teaching and learning among Saudi Arabian medical students.  The results of this study show the academic motivation section of the MSLQ has satisfactory internal consistency reliability. Contrary to exceptions, this study did not approve the hypothesised model of the academic motivation section of the MSLQ to be used for Saudi Arabian medical students. Alternatively stated, the model may not work for monitoring and improving teaching and learning in medicine.  Alternatively, our data sample may not represent the whole student given that female students are not included in this study. However, these results match those observed in the previous studies.^22^^, ^^35^^-^^38^  For example, in a study, using medical residents,  the researchers found that the original model fit poorly to their data.^22^  Furthermore, a poor fit was also found in another study that tested the validity of the questionnaire.^35^  It is difficult to explain these results, but it might be due to the fact that the educational and cultural contexts are different among Saudi Arabian medical students. Although the score reliability of the subscales of the academic motivation of the MSLQ is encouraging, there is abundant room for future research.

Finally, a number of important limitations need to be considered. First, the study is limited by the lack of data on female students.  Secondly, the data only came from a single institute. Therefore, the generalisability of these results is limited to the target population. Finally, the major limitation of this study is the low sample size.   These limitations mean that study results need to be interpreted cautiously.

## Conclusions

The present study was designed to measure the construct validity of the motivation section of the MSLQ and to assess its hypothesised model. The results of this study show the motivation section of the MSLQ is a reliable measure. However, the data do not fit the hypothesised model and therefore the questionnaire may not be valid. The reliability of scale score may encourage researchers to conduct further studies in order to obtain a clear picture of the MSLQ.  This research has generated some questions in need of future investigations.   For example, a future study with more focus on both genders is suggested. In addition to this, a large sample size could provide a clear picture of both sections of the MSQL for Saudi Arabian medical students.

### Conflict of Interest

The authors declare that they have no conflict of interest.

## Supplementary material

Supplementary file 1 Appendix 1. Students’ Motivation Scale

## References

[1] Pintrich P, Smith D, Garcia T, Mckeachie W (1993). Reliability and predictive validity of the motivated strategies for learning questionnaire.. Educational and Psychological Measurement.

[2] Kusurkar RA, Ten Cate TJ, van Asperen M, Croiset G (2011). Motivation as an independent and a dependent variable in medical education: A review of the literature.. Med Teach.

[3] Schunk D, Meece J, Pintrich P. Motivation in education: theory, research, and applications. Essex: Pearson Education Limited; 2014.

[4] Goodman S, Jaffer T, Keresztesi M, Mamdani F, Mokgatle D, Musariri M, Pires J, Schlechter A (2011). An investigation of the relationship between students' motivation and academic performance as mediated by effort.. South African Journal of Psychology.

[5] Duncan TG, McKeachie WJ (2005). The Making of the Motivated Strategies for Learning Questionnaire.. Educational Psychologist.

[6] Pintrich P (1988). A process-oriented view of student motivation and cognition.. New Directions for Institutional Research.

[7] Pintrich P (1988). Student learning and college teaching.. New Directions for Teaching and Learning.

[8] Pintrich, PR, Smith DA, Garcia T, McKeachie WJ. A manual for the use of the motivated strategies for learning questionnaire (MSLQ). Ann Arbor, MI: University of Michigan; 1991.

[9] Bandalos D, Finney S, Geske J (2003). A model of statistics performance based on achievement goal theory.. Journal of Educational Psychology.

[10] Zusho A, Pintrich PR, Coppola B (2003). Skill and will: The role of motivation and cognition in the learning of college chemistry.. Int J Sci Educ.

[11] Brookhart SM, Durkin DT (2003). Classroom assessment, student motivation, and achievement in high school social studies classes.. Applied Measurement in Education.

[12] Ommundsen  Y (2003). Implicit theories of ability and self-regulation strategies in physical education classes.. Educational Psychology.

[13] Rao N, Moely BE, Sachs J (2000). Motivational beliefs, study strategies, and mathematics attainment in high- and low-achieving Chinese secondary school students.. Contemp Educ Psychol.

[14] Vogt C. An account of women's progress in engineering: a social cognitive perspective. J Women Minor Scien Eng. 2003; 9(3-4):22.

[15] Hargis J (2001). Can students learn science using the Internet?. Journal of Research on Computing in Education.

[16] Hancock DR (2002). Influencing graduate students' classroom achievement, homework habits and motivation to learn with verbal praise.. Educational Research.

[17] Niemi H, Nevgi A, Virtanen P (2003). Towards self-regulation in web-based learning.. Journal of Educational Media.

[18] Cheang KI (2009). Effect of learner-centered teaching on motivation and learning strategies in a third-year pharmacotherapy course.. Am J Pharm Educ.

[19] Saks K, Leijen Ä, Edovald T, Õun K. Cross-cultural adaptation and psychometric properties of the Estonian version of MSLQ. Procedia - Social and Behavioral Sciences. 2015; 191:597-604.

[20] Feiz P, Hooman H, kooshki S. Assessing the motivated strategies for learning questionnaire (MSLQ) in Iranian students: construct validity and reliability. Procedia - Social and Behavioral Sciences. 2013;84:1820-1825.

[21] Ilker GE, Arslan Y, Demirhan GA. Validity and reliability study of the motivated strategies for learning questionnaire. Education Sciences: Theory & Practice. 2014;14(3): 829-833.

[22] Cook DA, Thompson WG, Thomas KG (2011). The motivated strategies for learning questionnaire: score validity among medicine residents.. Med Educ.

[23] McKeachie W, Pintrich P, Lin Y. Teaching learning strategies. Educational Psychologist. 1985;20(3):153-160.

[24] Cook DA, Beckman TJ (2006). Current concepts in validity and reliability for psychometric instruments: theory and application.. Am J Med.

[25] International Test Commission. International guidelines on test adaptation. [Cited 15 Feb 2016]; Available from: https://www.intestcom.org/files/guideline_ test_adaptation.pdf.

[26] Taylor R. Review of the motivated strategies for learning questionnaire (MSLQ) using reliability generalization techniques to assess scale reliability (Doctoral dissertation). Auburn: Auburn University; 2012.

[27] Olivari MG, Bonanomi A, Gatti E, Confalonieri E. Psychometric properties of the motivated strategies for learning questionnaire (MSLQ) among Italian high school students. 14th European Congress of Psychology; Milan, 7-10 July 2015. Micol Tummino, Martina Bollati, Martina Widmann; 2015.

[28] Hamilton RJ, Akhter S (2009). Construct validity of the motivated strategies for learning questionnaire. Psychol Rep.

[29] Allison P. Missing data techniques for structural equation modeling. J Abnorm Psycho. 2003;112(4):545-557. 10.1037/0021-843X.112.4.545 14674868

[30] Schafer JL, Graham JW (2002). Missing data: our view of the state of the art.. Psychol Methods.

[31] Brown T. Confirmatory factor analysis for applied research. New York: the Guilford Press; 2006.

[32] Hu L, Bentler PM (1999). Cutoff criteria for fit indexes in covariance structure analysis: conventional criteria versus new alternatives.. Structural Equation Modeling: A Multidisciplinary Journal.

[33] Hooper D, Coughlan J, Mullen MR. Structural equation modelling: guidelines for determining model fit. The Electronic Journal of Business Research Methods. 2008;6(1): 53 - 60.

[34] Tavakol M, Dennick R (2011). Making sense of Cronbach's alpha.. Int J Med Educ.

[35] Cho MH, Summers J. Factor validity of the motivated strategies for learning questionnaire (MSLQ) in asynchronous online learning environments. Journal of Interactive Learning Research. 2012;23(1): 5-28.

[36] Earley M, Wisneski R, Fasko D. A critical literature review of the motivated strategies for learning questionnaire (MSLQ). 12th Biennial Conference of the European Association for Research on Learning and Instruction. August 28- September 1, 2007; Budapest, University of Szeged.

[37] Lynch DJ. Motivational factors, learning strategies and resource management as predictors of course grades. College Student Journal. 2006;40:423-8.

[38] Watson M, Mcsorley M, Foxcroft C, Watson A (2004). Exploring the motivation orientation and learning strategies of first year university learners.. Tertiary Education and Management.

