# Supplementary material for: Validity of the motivated strategies for learning questionnaire in Saudi Arabia
Source: Int J Med Educ. 2018 Nov 30;9:309–15. doi: 10.5116/ijme.5bec.81cf (PMC6387767; doi:10.5116/ijme.5bec.81cf)
Supplement: Supplementary file 1 — Appendix 1. Students’ Motivation Scale [file ijme-9-309-S1.pdf]

Appendix 1.

Students' Motivation Scale

1 = Not at all true of me	2	3	4	5	6	7 = Very true of me

1. In a class like this, I prefer course material that really challenges me so I can learn new things.
2. If I study in appropriate ways, then I will be able to learn the material in this course.
3. When I take a test I think about how poorly I am doing compared with other students.
4. I think I will be able to use what I learn in this course in other courses.
5. I believe I will receive an excellent grade in this class.
6. I'm certain I can understand the most difficult material presented in the readings for this course.
7. Getting a good grade in this class is the most satisfying thing for me right now.
8. When I take a test I think about items on other parts of the test I can't answer.
9. It is my own fault if I don't learn the material in this course.
10. It is important for me to learn the course material in this class.
11. The most important thing for me right now is improving my overall grade point average, so my main concern in this class is getting a good grade.
12. I'm confident I can learn the basic concepts taught in this course.
13. If I can, I want to get better grades in this class than most of the other students.
14. When I take tests I think of the consequences of failing.
15. I'm confident I can understand the most complex material presented by the instructor in this course.
16. In a class like this, I prefer course material that arouses my curiosity, even if it is difficult to learn.
17. I am very interested in the content area of this course.
18. If I try hard enough, then I will understand the course material.
19. I have an uneasy, upset feeling when I take an exam.
20. I'm confident I can do an excellent job on the assignments and tests in this course.
21. I expect to do well in this class.
22. The most satisfying thing for me in this course is trying to understand the content as thoroughly as possible.
23. I think the course material in this class is useful for me to learn.
24. When I have the opportunity in this class, I choose course assignments that I can learn from even if they don't guarantee a good grade.
25. If I don't understand the course material, it is because I didn't try hard enough.
26. I like the subject matter of this course.
27. Understanding the subject matter of this course is very important to me.
28. I feel my heart beating fast when I take an exam.
29. I'm certain I can master the skills being taught in this class.
30. I want to do well in this class because it is important to show my ability to my family, friends, employer, or others.
31. Considering the difficulty of this course, the teacher, and my skills, I think I will do well in this class.
